# A report of 12 cases of congenital hepatic hemangioma and literature review

**DOI:** 10.3389/fped.2025.1453019

**Published:** 2025-04-28

**Authors:** Ying Wei, Zhihui Rong, Jinzhi Gao, Ling Chen

**Affiliations:** ^1^ Department of Pediatrics, Tongji Hospital, Tongji Medical College, Huazhong University of Science and Technology, Wuhan, China; ^2^ Department of Pediatrics, Shanxi Bethune Hospital, Third Hospital of Shanxi Medical University, Taiyuan, China

**Keywords:** congenital hepatic hemangioma, propranolol, surgical resection, percutaneous hepatic hemangioma embolization, congestive heart failure

## Abstract

**Objective:**

To investigate the clinical features, complications, diagnosis and management of congenital hepatic hemangiomas(CHHs).

**Methods:**

12 neonates of CHH admitted to our hospital in the past 10 years were retrospectively analyzed, and the clinical manifestations, auxiliary examination results, diagnosis and treatment methods, clinical efficacy andprognosis were reviewed.

**Results:**

In this study, 12 neonates with CHHs were reported. Among them, 8 cases underwent surgical treatment and recovered well postoperatively. 3 cases received routine pharmacological treatment, were gradually recovering. Only one case, presenting with giant CHH and congestive heart failure (CHF) at birth, failed initial pharmacological treatment and underwent percutaneous hepatic hemangioma embolization but died postoperatively.

**Conclusion:**

Large CHHs tend to be complicated with refractory congestive heart failure, likely due to tumor size and intra-tumor arteriovenous shunt. Propranolol is effective for CHHs with stable hemodynamics but has a slow onset of action, making it less suitable for cases complicated with CHF. Surgical resection is effective and recommended for large CHHs with stable hemodynamics, while percutaneous hepatic hemangioma embolization is advised for unstable cases.

## Introduction

Hemangioma, a benign vascular tumor in infants and young children, often occurs in the dermal papillary layer, and extends to the skin surface. Most of them appear about 2 weeks after birth, but 30% have appeared at birth, with a male-to-female ratio of 1:3 (1, 2). The pathogenesis remains unclear. Based on the understanding of the biology of these lesions, they are clinically classified into two different types, infantile hemangiomas (IHs) and congenital hemangiomas (CHs). Occuring from the first week to the fourth week after birth, IHs can be divided into three periods: proliferation period, regression period and regression completion period. Different from IHs, CHs are fully developed at birth and lack a postnatal proliferative period, which has long been considered a hallmark feature of CHs (3). In 2018, CHs are divided into three different subtypes by the International Society for the Study of Vascular Anomalies (ISSVA): rapidly involuting congenital hemangioma (RICH), noninvoluting congenital hemangioma(NICH) and partially involuting congenital hemangioma (PICH). These subtypes share the common histopathological features (4). However, a fourth subtype of CHs over the years has recently been reported by several studies, which exhibited unusual postnatal proliferation immediately after birth, followed by complete/nearly complete involution or partial involution (3). Endothelial cells in CHs are not stained with Glucose transport albumen (GLUT-1), which is an important marker used to differentiate CHs from IHs (5). Some studies suggested that at least 1 year follow-up is required to determine the subtype of hemangioma (4, 6).

Hemangiomas are typically solitary lesions, most commonly located on the head, neck or limbs, with sizes ranging from a few millimeters to several centimeters (7). While primarily involving the skin, they can also affect internal organs, with the liver being the second most commonly involved (8). They can also involve the eyes, central nervous system and oral mucosa (8). Congenital hepatic hemangioma(CHH) is a benign, high-flow vascular tumor that proliferates *in utero* and is fully grown at birth, with no increase in size after birth (9). According to the International Society for the Study of Vascular Anomalies (ISSVA) guidelines, hepatic hemangiomas are classified into three subtypes: focal, multiple, and diffuse (9, 10). While CHHs are generally focal and asymptomatic, giant cases sometimes may lead to severe complications such as intracranial bleeding, thrombocytopenia, hypofibrinogenemia, and high output heart failure. Among these, CHF, consumptive coagulopathy, and liver rupture are particularly life-threatening. Liver failure is also a rare but serious complication (11). Giant CHHs, defined as tumors larger than 4 cm in diameter, have an incidence of 0.64 per 10,000 and mortality rates ranging from 30% to 100% (12, 13). Although most CHs can resolve naturally without special treatment, active intervention is still needed for large or visceral hemangiomas with hemodynamic instability.

Despite numerous small studies have reported the clinical characteristics and treatment outcomes of CHHs, clear recommendations for managing complex CHHs, remain lacking. Therefore, this study aims to report the clinical characteristics of a group of CHHs, and analyze these characteristics, complications and treatments, in order to provide insights for future management strategies.

## Method

**Figure 1:**
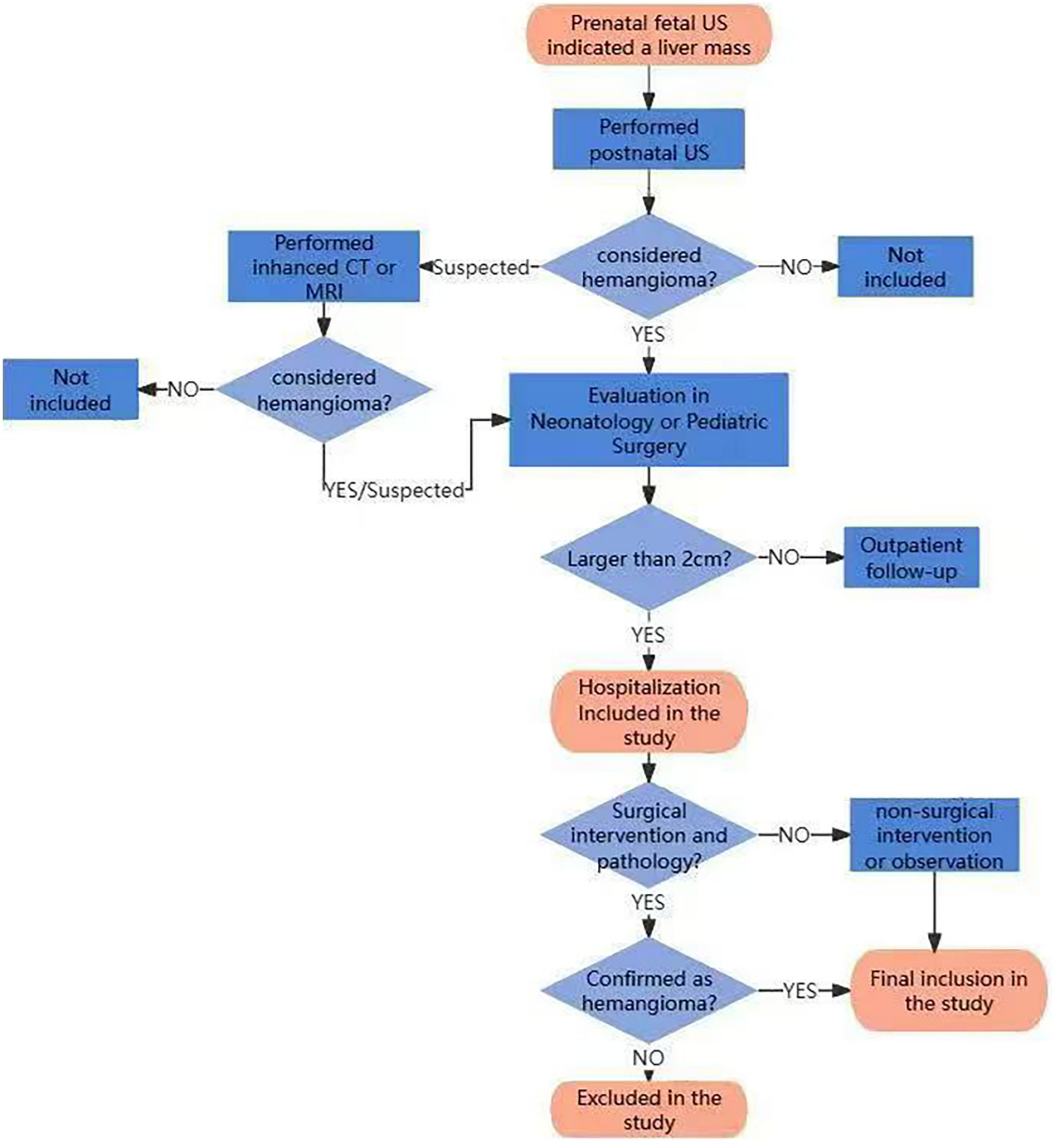
The flowchart of assessment, enrollment and exclusion for the patients.

All neonates diagnosed with CHHs who were hospitalized in the department of neonatology or pediatric surgery of Tongji Hospital, Tongji Medical College, Huazhong University of Science and Technology during 10 years from June 2014 to June 2023 were retrospectively collected. The diagnosis of CHHs was based on the classification system of the International Society for the Study of Vascular Anomalies (ISSVA). The diagnosis of CHH was established based on clinical features, radiological characteristics, and/or histopathological data. At our center, the management of CHHs was as follows: (1) Lesions ≤2 cm: Recommended outpatient monitoring with regular follow-up visits; (2) Lesions >2 cm: Hospital admission for comprehensive evaluation is indicated; (3) Lesions 2–4 cm: Treatment approach is determined by shared decision-making with parents/guardians, with options including medical therapy or active surveillance; (4) Lesions >4 cm: Management is individualized, with treatment options including surgical intervention or medical therapy based on comprehensive assessment. In case with complications, surgical intervention would be given priority; otherwise, medical therapy would be preferred as first-line approach. The flowchart of assessment, enrollment and exclusion for the patients after birth is shown in Figure 1.

Inclusion criteria: (1) The liver mass was detected by prenatal fetal ultrasound; (2) Postnatal liver imaging suggested the possibility of hemangioma, including US, CT or MRI scans of the liver; (3) The neonates had been hospitalized in the Department of Neonatology or Pediatric Surgery due to the size of the hepatic hemangioma greater than 2 cm, ensuring sufficient clinical data availability. All three criteria must be met for inclusion.

Exclusion criteria: 1. Clinical features and/or histopathological findings of the tumor lesion were inconsistent with CHH. Patients meeting the exclusion criteria were excluded from the study.

Clinical data, including prenatal ultrasound findings, clinical manifestations, diagnostic tests, treatment modalities, surgical details, pathology results, and prognosis, were collected from medical records.

## Result

**Figure 2:**
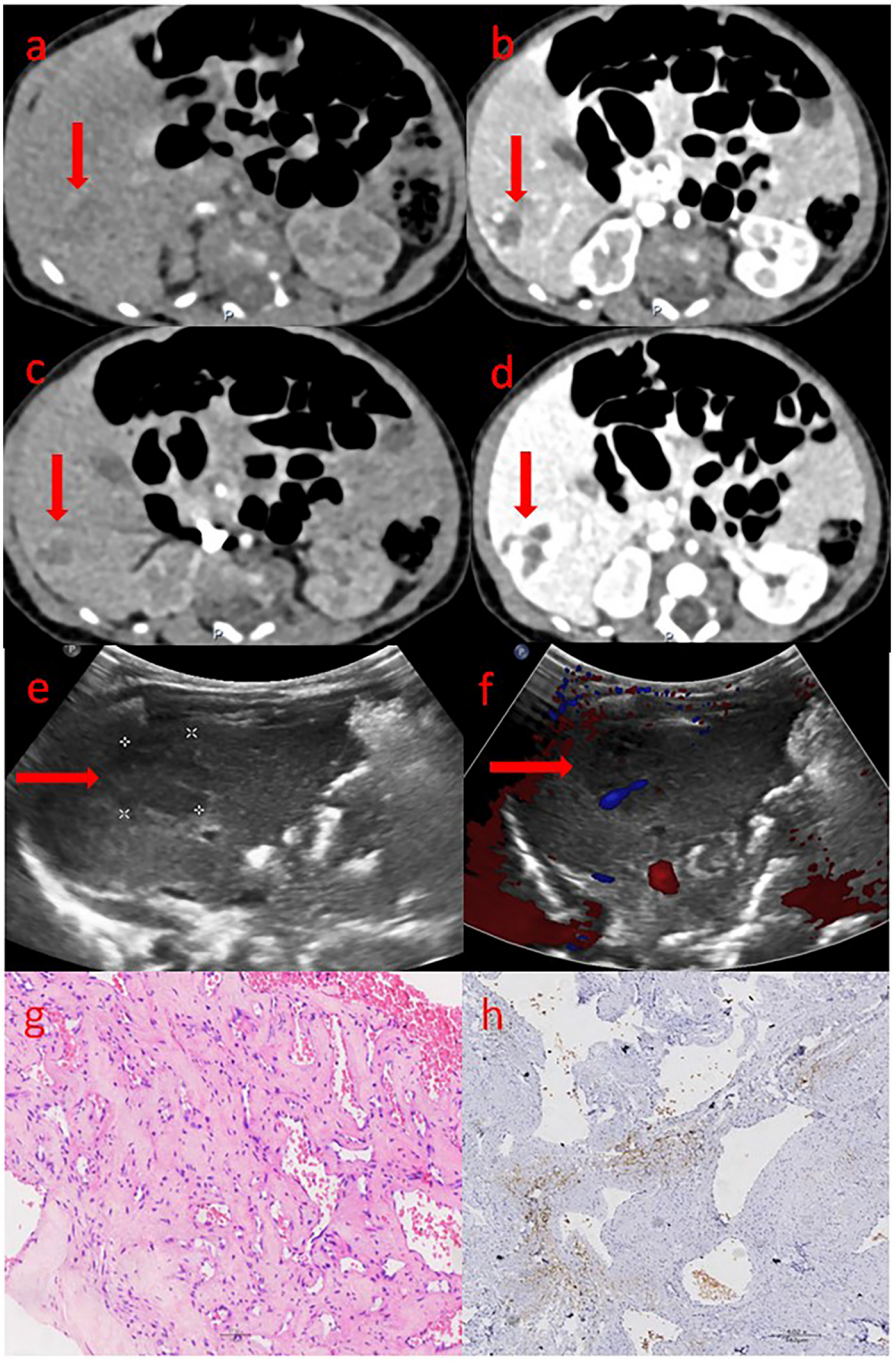
The ultrasound, CT and pathological images of congenital hepatic hemangioma. **(a–d)** shows the abdominal enhanced CT images: **(a)** Arterial phase; **(b)** Venous phase; **(c)** Portal phase; **(d)** Delay phase. The area marked by the arrow is the hemangioma. **(e,f)** shows the ultrasound images: **(e)** The two-dimensional gray-scale image; **(f)** The color Doppler image. The area marked by the arrow is the hemangioma. **(g,h)** shows the pathological images: **(g)** The tumor tissue shows varying degrees of vascular proliferation, dilation and congestion, as well as proliferation of fibrous tissue around the vessels. **(h)** endothelial cells do not express GLUT-1, red blood cells can be used as a positive internal control.

This study included 13 patients. These patients were found to have liver masses through prenatal fetal ultrasound. After birth, their liver masses were evaluated by liver ultrasound, CT or MRI, and were highly suspected or confirmed to be hemangiomas. The tumor sizes were all greater than 2 cm, and they received inpatient treatment or assessment in the neonatal department or pediatric surgery department. After excluding one case confirmed as liver hamartoma by postoperative pathology, a total of 12 cases were enrolled as CHHs, with 8 cases confirmed by pathology (Figures 2g,h) and 4 cases by imaging (Figures 2a–f).

As shown in Table 1, all 12 cases were full-term infants, with gestational ages ranging from 37 weeks +1 day to 40 weeks +1 day and birth weights between 2,650 and 3,900 g. Liver masses were detected prenatally by fetal ultrasound, with detection times ranging from 1 day to 14 weeks before birth. Except for case 12, which presented with thrombocytopenia and abnormal coagulation function, the other 11 cases had normal platelet counts and coagulation function. Case 1 was complicated with patent ductus arteriosus (PDA) and pulmonary hypertension, Case 6 by PDA, but the right heart function of the two cases remained normal. Case 12 was also complicated with PDA and pulmonary hypertension, but the baby already had right heart enlargement and right heart failure at birth. The remaining other cases did not exhibit any cardiac-related complications. Cases 1–8 underwent surgical resection of the lesion, with pathology confirming CHHs, and all recovered satisfactorily after operation. Cases 9–11 received pharmacological treatment (oral propranolol), and the tumor gradually shrank and finally disappeared during follow-up observation. Only Case 12, born with heart failure, initially received pharmacological treatment (oral propranolol and intravenous methylprednisolone). After the failure of pharmacological treatment, percutaneous embolization of hepatic hemangioma was performed, but the patient died on the second day after embolization.

**Table 1:** Clinical baseline data of 12 neonates with congenital hepatic hemangioma.

Clinical baseline data	Case 1	Case 2	Case 3	Case 4	Case 5	Case 6	Case 7	Case 8	Case 9	Case 10	Case 11	Case 12
Sex	Female	Female	Female	Male	Female	Male	Male	Male	Female	Male	Female	Female
Gestational age (w)	38 weeks + 3	38 weeks + 6	38 weeks + 1	38 weeks + 5	38 weeks + 2	40 weeks + 1	39 weeks	38 weeks	38 weeks	37 weeks + 1	38 weeks + 5	37 weeks + 4
Birth weight (g)	3,400	3,810	3,000	3,900	2,950	2,690	3,750	3,470	3,200	2,650	3,200	2,600
The first discovery time	4 weeks before delivery	6 weeks before delivery	1 day before delivery	4 days before delivery	2 days before delivery	2 days before delivery	1 week before delivery	5 days before delivery	4 days before delivery	1 week before delivery	3 days before delivery	14 weeks before delivery
Lesion size at birth on ultrasound (mm)	70*68	33*25	45*40	52*33	71*52	58*38	53*49	62*41	20*19	31*18	51*34	75*61
Other sites of hemangioma	No	No	No	No	No	No	No	No	No	No	Skin of head and upper abdomen	Right outer corner of eye, left hypothenar, left abdomen, left groin, left back and right lower extremity skin
PLT (*10^9 ^/L)	174	586	376	391	336	251	334	207	482	374	242	92
PT (s)	14.7	13.7	12.7	13.4	12.5	13.9	14.1	11.6	10.2	/	14.2	15.5
APTT (s)	41.3	41.6	51.8	51.7	37.5	46.1	49.3	44.6	49.8	/	40.8	64.6
FIB (g/L)	2.57	3.12	3.33	2.84	3.9	5.51	2.04	2.87	2.88	/	2.87	0.78
Cardiac uhrasonography	Patent ductus arteriosus, pulmonary hypertension	Normal	Normal	Normal	Normal	Patent ductus arteriosus	Normal	Normal	Normal	Normal	Normal	Patent ductus arteriosus, pulmonary hypertension, Right heart enlargement, right heart failure
Treatment method	Surgical excision	Surgical excision	Surgical excision	Surgical excision	Surgical excision	Surgical excision	Surgical excision	Surgical excision	Propranolol	Propranolol	Propranolol	Percutaneous embolization of hepatic hemangioma
Actual postoperative size (mm)	100*100*90	60*30*20	90*90*80	60*50*25	80*70*30	40*30	70*60*50	70*50*30	/	/	/	/
Pathology results	Hepatic congenital hemangioma	Hepatic congenital hemangioma	Hepatic congenital hemangioma	Hepatic congenital hemangioma	Hepatic congenital hemangioma	Hepatic congenital hemangioma	Hepatic congenital hemangioma	Hepatic congenital hemangioma	/	/	/	/
Prognosis	Fine	Fine	Fine	Fine	Fine	Fine	Fine	Fine	Gradually shrank and disappeared	Gradually shrank and disappeared	Gradually shrank and disappeared	Dead

**Figure 3:**
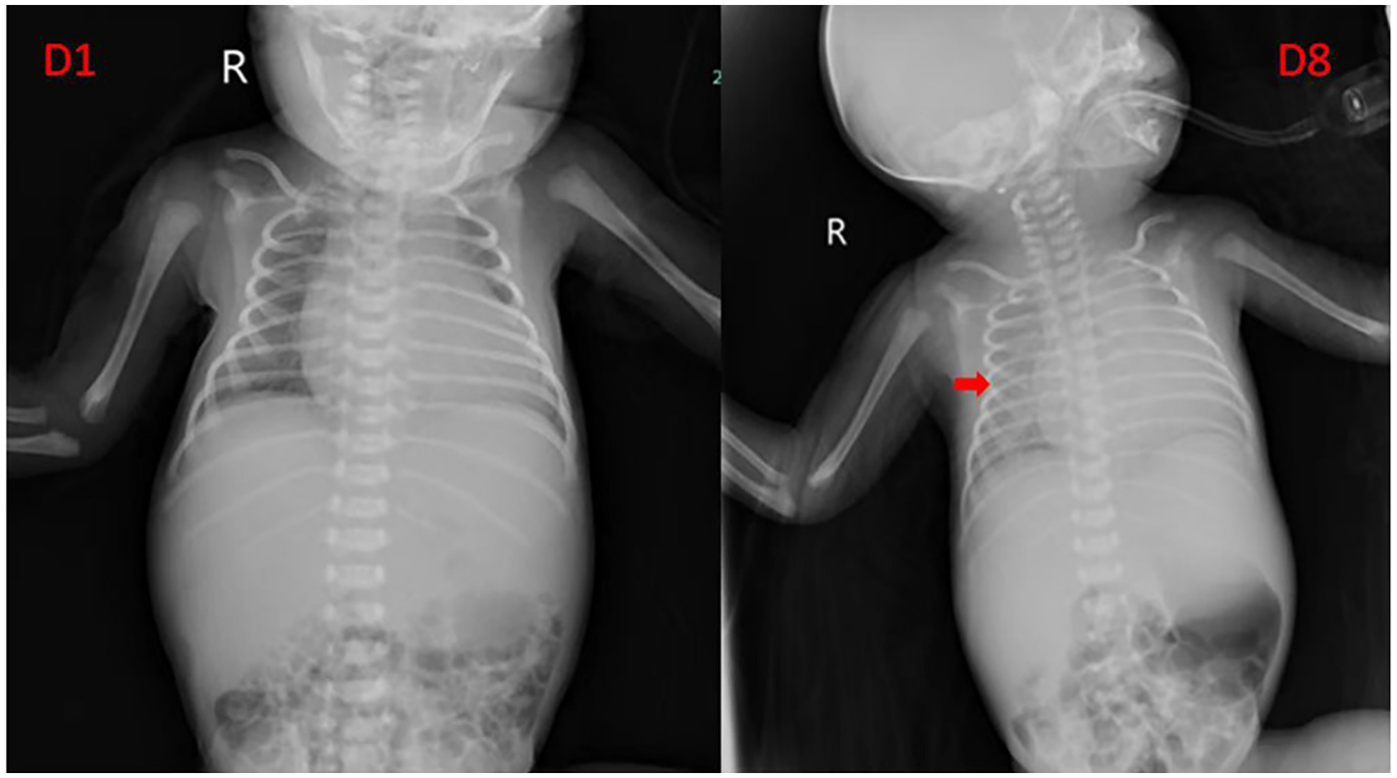
Comparison of chest radiograph before and after pharmacological treatment in case 12. The arrow indicates that the transparency of both lungs is uniformly decreased, suggesting pulmonary edema.

**Figure 4:**
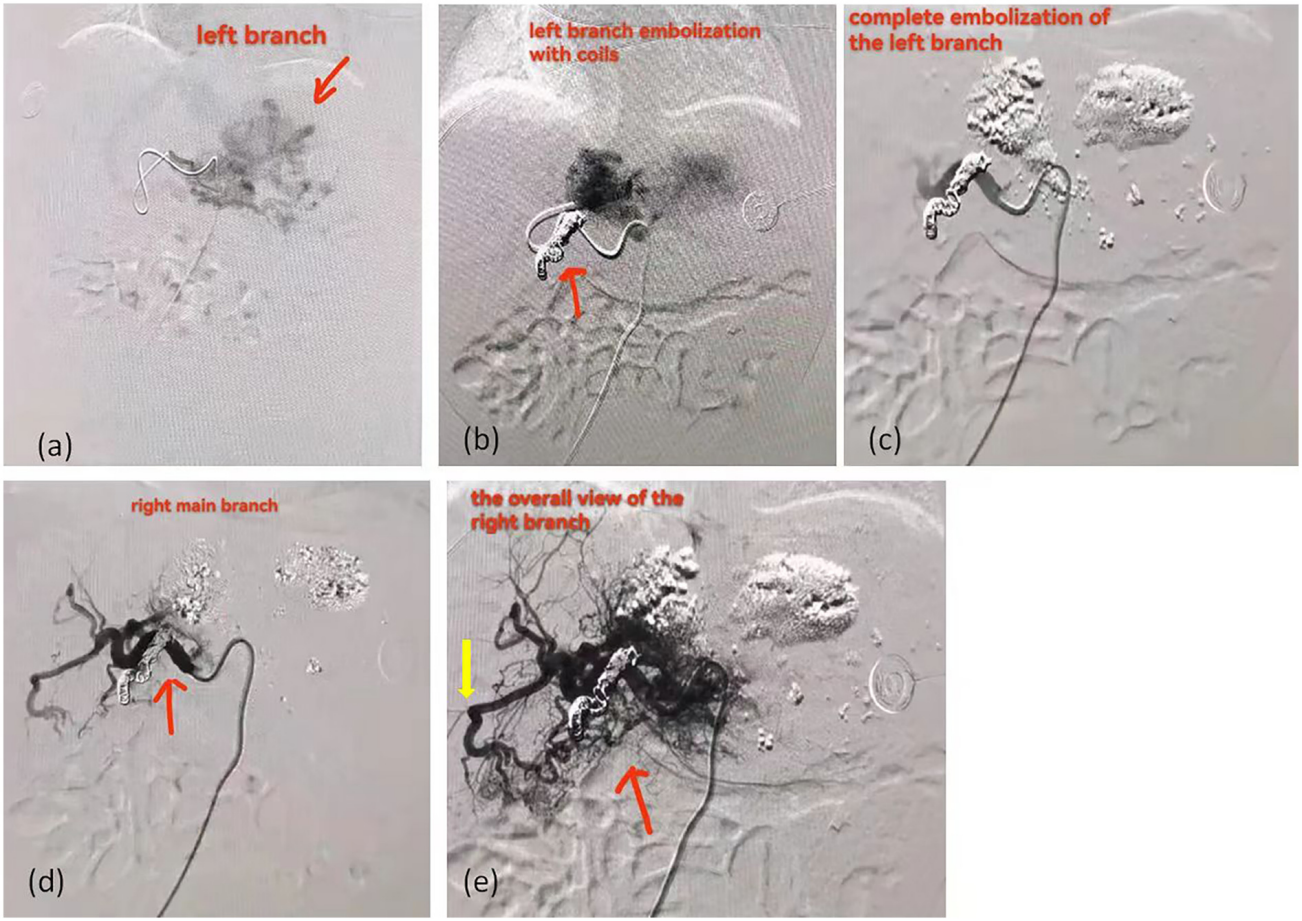
The angiographic images of case 12 received percutaneous hepatic hemangioma embolization. **(a)** The left branch of the hemangioma. The area marked by the arrow is the hemangioma. **(b)** The image after embolization of the left main trunk with coils show that there are still hazy malformed vessels at the distal end. The area marked by the arrow is the coils. **(c)** The image after filling the fine vascular network with ultra-liquefied iodized oil, pingyangmycin, and gelatin sponge particles. **(d)** The right main trunk of the hemangioma. The area marked by the arrow is the right main trunk. **(e)** The overall view of right branch of the hemangioma. The red arrow indicates the overall view of right branch; The yellow arrow indicates the arteriovenous shunt.

As shown in Table 2, Case 12 presented with heart failure and abnormal coagulation function at birth. Postnatal management included fluid restriction, inotropic support (cedilan), and diuresis to alleviate heart failure, as well as multiple infusions of fibrinogen and fresh frozen plasma to improve coagulation function. Meanwhile, the patient was also treated with propranolol (starting at 0.5 mg/kg.d and gradually increasing to 2 mg/kg.d) and methylprednisolone for pharmacological treatment. One week later, the patient's condition worsened, with increased brain natriuretic peptide (BNP) levels, deteriorated coagulation function, and aggravated dyspnea. As shown in Figure 3, the comparison of chest radiographs revealed significantly worsened pulmonary edema compared to the initial presentation. Respiratory support was escalated from high-flow nasal cannula at admission to invasive mechanical ventilation. Therefore, percutaneous hepatic hemangioma embolization was performed on the 9th day after birth. The multidisciplinary team, consisting of interventional radiologists, neonatologists, pediatric cardiologists, pediatric surgeons, and anesthesiologists, collaboratively participated in the decision-making and intraoperative management. The embolization procedure began with angiography to assess the morphology and branching of the hemangioma. The angiography revealed two large malformed vessels with arteriovenous fistulas, followed by a cloud-like network of fine vessels, as shown in Figures 4a–e. Based on these findings, the multidisciplinary team unanimously decided to embolize the large left branches using microcoils (Figure 4b) combined with gelatin sponge particles, while filling the fine vascular network with ultra-liquefied iodized oil, pingyangmycin, and gelatin sponge particles (Figure 4c). During the procedure, the larger branch on the left side and its tributaries were successfully embolized (Figure 4c). However, difficulty was encountered in accessing the large branch on the right side (Figures 4d,e). After two unsuccessful attempts and considering that the intervention had already lasted one hour, the team decided to conclude the procedure, with plans for staged re-embolization or surgical intervention at a later time. Unfortunately, on the second postoperative day, Case 12 experienced sudden cardiac arrest and died despite resuscitation efforts. Unfortunately, the parents signed off on the autopsy, and we don't know the true cause of the child's cardiac arrest.

**Table 2:** Comparison of the clinical parameters in case 12 before and after pharmacological treatment.

Clinical parameters	Before pharmacological treatment (D1)	After pharmacological treatment (D8)
TnI (pg/ml)	674.2 (<15.6)	671.9 (<15.6)
NT-ProBNP (pg/ml)	45,759 (<1,000)	54,867 (<1,000)
PLT (*10^9 ^/L)	92 (150–300)	89 (150–300)
PT (s)	15.2 (9.5–12.5)	13.8 (9.5–12.5)
APTT (s)	53.2 (28.0–43.0)	64.6 (28.0–43.0)
Fib (g/L)	1.34 (1.5–3.0)	0.91 (1.5–3.0)
D-D (ug/ml FEU)	21.64 (<0.5)	4.58 (<0.5)
The lesion size on liver ultrasound (cm)	7.5 × 6.1	7.0 × 6.2
chest radiography	Slightly reduced opacity in both lungs	Uniformly and significantly decreased transparency of both lungs, suggesting pulmonary edema
Respiratory support method	Nasal high flow assisted ventilation	Invasive ventilator-assisted ventilation

## Discussion

In this study, 12 cases with CHHs were reported. 8 cases underwent surgical treatment and achieved satisfactory recovery after surgery. 3 cases received routine pharmacological treatment, and the tumor gradually shrank and finally disappeared during follow-up observation. One case, complicated by CHF at birth, underwent percutaneous embolization of hepatic hemangioma after failure of initial pharmacological treatment and died on the second day after embolization.

Studies have reported that CHF seems to be more likely to occur in cases of visceral hemangioma or multiple hemangioma, while in cutaneous CHs, CHF appears to correlate with tumor size, especially larger than 7 cm in diameter (7, 14). In this study, 9 cases were all diagnosed with giant CHHs, 3 of which had tumors larger than 7 cm postoperatively. However, only 1 patient (case 12) developed CHF and right heart enlargement. This patient had a tumor measuring 7.5 × 6.1 cm at birth, along with hemangiomas in multiple extracutaneous sites, including the outer corner of the right eye, the left hypothenar, the left abdomen, the left groin, the left back and the right lower limb, which was consistent with the above studies. Some studies have suggested that the pathogenesis of CHF may be related to extensive arteriovenous shunt and venous lake in hemangioma tissue (15). Extensive arteriovenous shunt creates a phenomenon of “blood stealing” that puts other peripheral tissues at risk of infarction. In addition, arteriovenous shunt reduces peripheral circulatory resistance, resulting in a hyperdynamic state of circulation that worsens myocardial perfusion and may lead to progressive myocardial ischemia, while also contributing to the development of heart failure by increasing pulmonary backflow (15). In this study, the liver angiography of case 12 revealed multiple arteriovenous shunt vessels with coarse malformations and venous lakes. The case was born with heart failure and right heart enlargement, and lost the opportunity for surgery. However, the liver ultrasonography in other cases with giant CHHs showed no evidence of arteriovenous shunt and did not develop CHF, further supporting this hypothesis.

Although most CHs are uncomplicated and do not require active treatment in the neonatal period, complex CHs with serious complications, especially with CHF, may have a very high mortality rate, indicating the need for early close monitoring and active intervention (12). Current treatment strategies for CHs are largely refered from those for IHs. Reported drug therapies for IHs include corticosteroids, sirolimus and propranolol (16, 17). Propranolol is currently the only FDA-approved drug for IH (8). However, no prospective research data has been published to confirm the effectiveness and safety of various drugs in the treatment of IHs, and corresponding clinical guidelines and consensus remain lacking, leading to potential bias in existing data (17, 18).

Systemic steroids have been the primary and most commonly used treatment for hemangiomas (8). Studies on IHs show that corticosteroids inhibit the angiogenesis of infantile-hemangioma-derived stem cells by inhibiting vascular endothelial growth factor A (VEGF-A). Early proliferative IHs, which contain a larger proportion of stem cells, are more easily inhibited by corticosteroids than late proliferative IHs (8). However, CHs proliferate *in utero* and lack a postnatal proliferative period, so it is speculated that corticosteroids may have a limited therapeutic effect in CHs (12).

As early as 2008, propranolol has become a first-line treatment for IHs (17, 19–21). Propranolol is a synthetic β-adrenergic receptor blocker, the mechanism of action on IHs is not very clear. Some proposed hypotheses include vasoconstriction, decreased renin production, inhibition of angiogenesis, and induction of apoptosis (8). Given propranolol's ability to induce apoptosis, it may be more effective than corticosteroids for CHs. In this study, the masses of the three patients who received pharmacological treatment gradually shrank and eventually disappeared during follow-up observation. Some studies have indicated that the apoptotic effect of propranolol may take 12–14 months to take effect, limiting its utility in life-threatening complications (7). In cases with CHs complicated with CHF, clinicians must balance the risk of bradycardia and possible myocardial inhibition with the potential to reduce tumor size when using propranolol. Propranolol must be used with caution in infants with poor compensation or acute CHF (7). The patient's clinical parameters and hemodynamic stability should guide treatment.

The mammalian target of rapamycin (mTOR) is associated with cell proliferation and angiogenesis, and its inhibitors can suppress these processes. Both sirolimus and everolimus, as mTOR inhibitors, have demonstrated anti-proliferative and anti-angiogenic effects in *in vitro* studies of hemangioma endothelial cells, suggesting their potential role in the treatment of infantile hemangiomas(IHs) (22). Several case reports have documented the use of mTOR inhibitors in IHs, but these are mostly isolated cases (22–24). A recent systematic review of 73 studies that included 373 patients indicated that sirolimus showed promising results in the management of various vascular anomalies (13). Further research is needed to confirm their efficacy and safety in the treatment of congenital hemangiomas (CHs).

Embolization may also be effective alternative when rapid symptom relief is needed (25). Although embolization cannot remove hemangioma, it can quickly relieve symptoms, making it a valuable adjunct for severe HHs complicated by rupture, CHF, or large shunts, especially when propranolol fails (9, 25). The liver's dual blood supply (30% from the hepatic artery and 70% from the portal vein) and the potential for multiple feeding vessels of the tumors complicate the identification and embolization of the primary supply artery. In addition, the risk of small blood vessels, limited use of contrast agent and massive blood loss make embolization particularly challenging in infants. However, this technique is often a complex and invasive operation that requires an interventional therapist with specialized skills and knowledge to complete. It has been reported that the detachable coils and α-N butyl cyanoacrylate (NBCA) adhesive can be used to perform intravascular embolization for the treatment of CHHs in children with CHF (14). NBCA adhesive has the advantages of low viscosity, fast curing speed, strong permeability, low tissue toxicity and no dependence on coagulation function. However, special attention should be paid to the possibility of the NBCA adhesive shingling into the lungs leading to iatrogenic pulmonary embolism (14). Gelatin sponge has also been reported to have successfully achieved embolization of CHH (26). In this study, Case 12 presented with severe complications at birth, including heart failure and abnormal coagulation function. Considering the risks associated with surgery and anesthesia, pharmacological treatment was initially administered. However, due to the progression of congestive heart failure (CHF), complicated by pulmonary edema and respiratory failure, percutaneous hepatic hemangioma embolization was ultimately chosen. As our center is a general hospital primarily serving adults, infantile and congenital hepatic hemangiomas are relatively rare. Therefore, the embolization procedure was led by an interventional radiologist with extensive experience in treating adult hepatic hemangiomas, supported by a multidisciplinary team including neonatologists, pediatric cardiologists, pediatric surgeons, and anesthesiologists. The choice of embolic agents was based on the interventional radiologist's experience with adult cases. Microcoils combined with gelatin sponge particles were used to embolize the large branches, while ultra-liquefied iodized oil, pingyangmycin, and gelatin sponge particles were employed to fill the fine vascular network. Unfortunately, the patient experienced sudden cardiac death postoperatively. A multidisciplinary mortality review suggested that the gelatin sponge particles (a particulate embolic agent) and iodized oil (a liquid embolic agent) might have migrated through arteriovenous shunts to the lungs or coronary arteries, potentially causing myocardial infarction or iatrogenic pulmonary embolism, particularly in the context of high-output CHF.

In some cases, surgical intervention may be necessary, especially in the face of complications such as uncontrolled intratumoral bleeding (27). However, surgery for giant HHs is technically challenging and carries significant risks. The size, location and quantity of the tumor and the relationship between the tumor and neighboring tissues must be considered during the operation. In particular, the huge HHs in the hepatic portal area may cause massive bleeding during the operation, endangering the life of the patient (28). The key to the success of the operation is to choose a reasonable surgical method and control the amount of bleeding during the operation. In this study, 8 cases with giant CHHs at birth were surgically removed the liver segment under stable hemodynamic conditions, with favorable outcomes. When drug therapy and embolization fail or surgical local resection is not feasible, complete hepatectomy may be considered as soon as possible, followed by liver transplantation, but with a higher mortality rate. Therefore, total hepatectomy and liver transplantation should only be used as a last resort (9).

Finally, this study has some limitations, such as a retrospective study with a small sample size, which may lead to inevitable uncertainty bias. Nevertheless, our findings suggest that giant CHHs are prone to refractory CHF, likely due to tumor size and intratumoral arteriovenous shunting. Propranolol is effective for CHHs with stable hemodynamics but should be used cautiously in cases complicated by CHF. Surgical resection is recommended for large CHHs with stable hemodynamics, while percutaneous hepatic hemangioma embolization is advised for unstable cases. In short, the optimal management of complex CHHs requires multidisciplinary cooperation involving neonatology, pediatric cardiology, neonatal surgery, anesthesiology and vascular interventional radiology.

## Data Availability

The original contributions presented in the study are included in the article/Supplementary Material, further inquiries can be directed to the corresponding author.

## Supplementary material

The Supplementary Material for this article can be found online at: https://www.frontiersin.org/articles/10.3389/fped.2025. 1453019/full#supplementary-material
