# Corrigendum: A report of 12 cases of congenital hepatic hemangioma and literature review

**DOI:** 10.3389/fped.2025.1629270

**Published:** 2025-06-09

**Authors:** Ying Wei, Zhihui Rong, Jinzhi Gao, Ling Chen

**Affiliations:** ^1^ Department of Pediatrics, Tongji Hospital, Tongji Medical College, Huazhong University of Science and Technology, Wuhan, China; ^2^ Department of Pediatrics, Shanxi Bethune Hospital, Third Hospital of Shanxi Medical University, Taiyuan, China

**Keywords:** congenital hepatic hemangioma, propranolol, surgical resection, percutaneous hepatic hemangioma embolization, congestive heart failure

Corrigendum on A report of 12 cases of congenital hepatic hemangioma and literature review By Wei Y, Rong Z, Gao J and Chen L (2025). Front Pediatr. 13:1453019. doi: 10.3389/fped.2025.1453019

**Additional Affiliation(s)**

In the published article, there was an error regarding the affiliation(s) for [Ling Chen]. As well as having affiliation(s) [1. Department of Pediatrics, Tongji Hospital, Tongji Medical College, Huazhong University of Science and Technology, Wuhan, China], they should also have [2. Department of Pediatrics, Shanxi Bethune Hospital, Third Hospital of Shanxi Medical University, Taiyuan, China].

The authors apologize for this error and state that this does not change the scientific conclusions of the article in any way. The original article has been updated.

